# Radiation and chemotherapy for high‐risk lower grade gliomas: Choosing between temozolomide and PCV

**DOI:** 10.1002/cam4.2686

**Published:** 2019-11-07

**Authors:** Susan G. R. McDuff, Jorg Dietrich, Katelyn M. Atkins, Kevin S. Oh, Jay S. Loeffler, Helen A. Shih

**Affiliations:** ^1^ Department of Radiation Oncology Duke Cancer Center, Medicine Circle Durham NC USA; ^2^ Department of Neurology Division of Neuro‐Oncology Massachusetts General Hospital Boston MA USA; ^3^ Department of Radiation Oncology Cedars‐Sinai Medical Center Los Angeles CA USA; ^4^ Department of Radiation Oncology Massachusetts General Hospital Boston MA USA

**Keywords:** chemotherapy, low grade glioma, PCV, radiotherapy, temozolomide

## Abstract

**Purpose:**

The majority of patients with high‐risk lower grade gliomas (LGG) are treated with single‐agent temozolomide (TMZ) and radiotherapy despite three randomized trials showing a striking overall survival benefit with adjuvant procarbazine, lomustine, and vincristine (PCV) chemotherapy and radiotherapy. This article aims to evaluate the evidence and rationale for the widespread use of TMZ instead of PCV for high‐risk LGG.

**Methods and Materials:**

We conducted a literature search utilizing PubMed for articles investigating the combination of radiotherapy and chemotherapy for high‐risk LGG and analyzed the results of these studies.

**Results:**

For patients with *IDH* mutant 1p/19q codeleted LGG tumors, there is limited evidence to support the use of TMZ. In medically fit patients with codeleted disease, existing data demonstrate a large survival benefit for PCV as compared to adjuvant radiation therapy alone. For patients with non‐1p/19q codeleted LGG, early data from the CATNON study supports inclusion of adjuvant TMZ for 12 months. Subset analyses of the RTOG 9402 and EORTC 26951 do not demonstrate a survival benefit for adjuvant PCV for non‐1p/19q codeleted gliomas, however secondary analyses of RTOG 9802 and RTOG 9402 demonstrated survival benefit in any *IDH* mutant lower grade gliomas, regardless of 1p/19q codeletion status.

**Conclusions:**

At present, we conclude that current evidence does not support the widespread use of TMZ over PCV for all patients with high‐risk LGG, and we instead recommend tailoring chemotherapy recommendation based on *IDH* status, favoring adjuvant PCV for patients with any *IDH* mutant tumors, both those that harbor 1p/19q codeletion and those non‐1p/19q codeleted. Given the critical role radiation plays in the treatment of LGG, radiation oncologists should be actively involved in discussions regarding chemotherapy choice in order to optimize treatment for their patients.

## Graphical abstract

Three randomized trials have shown a striking overall survival benefit with adjuvant procarbazine, lomustine, and vincristine (PCV) chemotherapy and radiotherapy for the treatment of patients with high‐risk lower grade gliomas, however the majority of patients currently receive temozolomide (TMZ) instead of PCV chemotherapy. This review article examines the best available evidence to inform the choice of PCV or TMZ for the treatment of high‐risk lower grade gliomas.

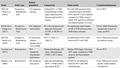

## 1 INTRODUCTION

High‐risk lower‐grade gliomas (LGG) constitute a heterogeneous group of tumors arising from glial cells (astrocytes and oligodendrocytes) in the central nervous system (CNS), often afflicting otherwise healthy young adults while exhibiting a more indolent course compared to adults with glioblastoma.^1^ Historically, World Health Organization (WHO, 2007) grade IIIII gliomas were classified histologically as astrocytomas, oligodendrogliomas, or mixed oligoastrocytomas, based on histopathology alone. However, in 2016, the WHO issued an updated classification for primary CNS tumors utilizing molecular parameters in addition to histology.^2^, ^3^, ^4^ The distinction of grade II vs grade III has given way to *IDH* mutation status foremost and then 1p/19q codeletion. Notably, *IDH* status does not play a role in grading and 1p/19q codeletion automatically renders a tumor an oligodendroglioma. Herein, we refer to “high‐risk lower grade gliomas (LGG)” as a category encompassing patients who were largely WHO 2016 *IDH1* mutant anaplastic gliomas (WHO 2007 grade III anaplastic oligodendroglioma or anaplastic mixed oligoastrocytoma) and grade II gliomas of any histology with unfavorable features (eg, age > 40, subtotal resection, tumor crossing midline).^5^, ^6^

While observation following surgical resection is acceptable for asymptomatic favorable‐prognosis patients, there has historically been controversy surrounding the optimal timing for adjuvant radiotherapy and chemotherapy for patients with high‐risk lower grade gliomas.^7^, ^8^, ^9^ Three randomized trials, including recently reported long‐term results from the landmark Radiation Therapy Oncology Group (RTOG) 9802 study, have shown a striking overall survival (OS) benefit with the addition of procarbazine, lomustine (CCNU), and vincristine (PCV) chemotherapy and radiotherapy for the treatment of patients with high‐risk lower grade gliomas.^5^, ^10^, ^11^ However, given difficulties with administration and tolerability of PCV, many neuro‐oncologists and medical oncologists frequently substitute the alkylating and oral agent temozolomide (TMZ) in place of multi‐agent chemotherapy in this setting.^12^, ^13^ In fact, the vast majority of patients with high‐risk LGG treated with radiation and chemotherapy receive single‐agent instead of multi‐agent chemotherapy in the United States (>95%),^14^ despite strong level‐one evidence supporting the use of PCV and the absence of any randomized studies comparing PCV vs TMZ in this setting. ALLIANCE‐N0577‐CODEL is an ongoing prospective trial aiming to resolve this controversy by randomizing patients with 1p/19q codeleted tumors (WHO grade III anaplastic gliomas or high‐risk WHO grade II low grade gliomas) to receive adjuvant PCV following radiotherapy vs concurrent and adjuvant TMZ, however, mature results are not expected for another 7‐10 years.^15^

In the interim, physicians are faced with the challenge of whether to administer PCV or the more tolerable TMZ to patients with unfavorable prognosis LGG. Thus, we performed a literature review to determine whether there is sufficient data to support the widespread use of TMZ instead of PCV in the treatment of patients with high‐risk LGG.

## 2 ADJUVANT PCV IMPROVES OVERALL SURVIVAL AND PROGRESSION FREE SURVIVAL FOLLOWING RADIOTHERAPY FOR LOWER GRADE GLIOMAS

To date, three major randomized trials have demonstrated a survival advantage among patients treated with adjuvant PCV compared to radiotherapy alone after resection of LGG.^5^, ^10^, ^11^ In the landmark RTOG 9802 study, 251 patients with newly diagnosed supratentorial WHO grade II astrocytoma, oligodendroglioma, or oligoastrocytoma with unfavorable features as defined by age ≥ 40 and/or subtotal resection (STR) were enrolled between 1998 and 2002 and randomized to receive radiotherapy alone or radiotherapy followed by six, 8‐week cycles of PCV chemotherapy.^5^, ^16^ Histologic review was required prior to randomization. Radiation therapy in both arms consisted of 54 Gy delivered in 1.8‐Gy daily fractions prescribed to isocenter. The target included areas of T2/FLAIR hyperintensity on magnetic resonance imaging (MRI) plus a 2cm margin to block edge.^16^ Patients in the radiotherapy with PCV arm were prescribed standard dosing of 110 mg/m^2^ lomustine (day 1), procarbazine 60 mg/m^2^ (days 8‐21), and vincristine 1.4 mg/m^2^ (2.0 mg maximum, days 8 and 29). Post hoc molecular subtyping of 113 cases revealed that *IDH1‐R132H* mutations were present in 61% of the group that received radiotherapy alone (35 of 57 patients) and 64% of the group that received radiotherapy with PCV (36 of 36 patients).^5^ 1p/19q codeletion status was assessable in 63 cases: 29 patients in the radiotherapy alone arm and 34 patients in the radiotherapy with PCV arm.^5^ At a median follow‐up of 11.9 years, patients randomized to receive PCV after radiotherapy had improved progression free survival (PFS) compared to those randomized to receive radiotherapy alone (median PFS 10.4 vs 4.0 years, respectively, HR 0.50, *P* < .001). Additionally, patients in the PCV arm exhibited improved OS compared to those in the radiotherapy alone arm (median OS 13.3 years vs 7.8 years, respectively, HR 0.59, *P* = .003). Notably, the difference in OS between treatment arms emerged after 3‐5 years of follow‐up.

In an early exploratory analysis of RTOG 9802, the subgroup of patients with oligodendrogliomas showed the greatest benefit statistically in terms of benefit from PCV whereas the astrocytoma subgroup only showed a trend (but did not reach statistical significance). Similarly, patients with tumoral *IDH1‐R132H* mutations who received radiotherapy and PCV had longer OS compared to those who received radiotherapy alone (but initially there were too few events to assess the impact of treatment among those with *IDH* wild‐type status).^5^ More recently, molecular profiling was possible in 106/251 (41%) of the RTOG 9802 cohort, and a post hoc subgroup analysis of outcomes as a function of *IDH* and 1p/19q codeletion status was reported at ASTRO 2019.^17^ As expected, median survival times differed as a function of WHO 2016 prognostic categories, with a median survival of 1.9 years among those with *IDH*‐wild type tumors, 6.9 years for those with *IDH*‐mutant/non‐codeleted tumors, and 13.9 years for those with *IDH*‐mutant/co‐deleted tumors.^17^ Interestingly, both *IDH*‐mutant subgroups demonstrated a survival benefit with the addition of PCV (4.3 vs 11.4 years for patients with *IDH*‐mutant/noncodeleted tumors, and 13.9 years vs not reached for patients with *IDH*‐mutant/codeleted tumors), while the *IDH*‐wild‐type group did not derive a benefit from PCV (median survival 1.9 vs 2.1 years).^17^

The European Organization for Research and Treatment of Cancer (EORTC) 26 951 study randomized 368 patients between 1995 and 2002 with newly diagnosed anaplastic oligodendroglial tumors to receive either radiotherapy alone or radiotherapy followed by 6 cycles of adjuvant PCV with standard dosing and a 2.0 mg maximum vincristine limit.^10^, ^18^ Molecular subtyping performed on 316 available cases revealed 80 (25%) with 1p/19q codeletion, which was reported to be balanced across treatment arms.^10^, ^18^ Radiotherapy was prescribed to a total dose of 59.4 Gy in 1.8 Gy daily fractions, including 45 Gy delivered to preoperative areas of low‐density on computed tomography (CT) scan or high‐density on T2‐weighted MRI with a 2.5 cm margin followed by a 14.4 Gy boost to the postoperative enhancing region on CT or MRI. At a median follow‐up of 140 months, patients who received radiotherapy followed by adjuvant PCV exhibited significantly longer OS, 42.3 months compared to 30.6 months (HR 0.75, *P* = .018), as well as longer PFS of 24.3 months vs 13.2 months (HR 0.66, *P* = .003).^10^ On post hoc analysis, survival benefit of PCV was more pronounced among patients with molecular 1p/19q codeletion, and patients without 1p/19q codeletion did not exhibit a survival benefit with adjuvant PCV.^10^

In the RTOG 9402 trial, 291 patients with anaplastic oligodendroglial tumors were randomized between 1994 to 2002 to receive intensive PCV followed by radiotherapy vs radiotherapy alone.^11^, ^19^ Post hoc molecular subtyping was available for 263 patients and revealed that 126 patients overall (48%) harbored a 1p/19q codeletion: 59 (44%) of 135 in the PCV plus RT arm and 67 (52%) of 128 in the RT arm.^11^ Patients in the intensive PCV and radiotherapy arm received 4 cycles of PCV every 6 weeks before radiotherapy with the following escalated dosing: 130 mg/m^2^ lomustine (day 1), procarbazine 75 mg/m^2^ (days 8 to 21), and vincristine 1.4 mg/m^2^ (with no 2 mg upper dose limit, days 8 and 29).^11^ Radiotherapy in both arms was prescribed to a total dose of 59.4 Gy in 1.8 Gy daily fractions, including an initial field delineated by the T2‐weighted abnormality plus a 2 cm margin, followed by a 9 Gy boost to the contrast enhanced T1‐weighted abnormality plus a 1 cm margin. Patients who received PCV and radiotherapy experienced superior PFS compared to those receiving radiotherapy alone (2.6 years vs 1.7 years, *P* = .004).^19^ On a longer follow‐up post hoc analysis, patients with 1p/19q codeleted tumors experienced significantly longer OS following treatment with PCV and radiotherapy compared to radiation therapy alone (14.7 vs 7.3 years, *P* = .03), whereas this benefit was not seen among patients without 1p/19q codeleted tumors.^11^ A subsequent analysis demonstrated that patients in this study with *IDH*‐mutant gliomas experienced significantly prolonged survival after treatment with PCV and radiotherapy compared to radiotherapy alone.^20^ The benefit provided by the addition of PCV was seen for both patients with *IDH*‐mutant codeleted tumors (14.7 vs 6.8 years, *P* = .01) and *IDH*‐mutant noncodeleted tumors (5.5 vs 3.3 years, *P* < .05).^20^

Taken together, these three randomized trials provide strong evidence supporting the use of adjuvant PCV chemotherapy in conjunction with radiotherapy for patients with high‐risk LGG, particularly those harboring 1p/19q codeletion. Importantly, however, the vast majority of patients continue to receive TMZ in place of PCV (>95%) in the United States. Why is this the case?

## 3 PRACTICAL LIMITATIONS OF PCV CHEMOTHERAPY

One reason to explain the substitution of PCV for TMZ in the management of high‐risk LGG is the relative difficulty of administering intravenous vincristine and the greater toxicity of PCV compared to TMZ.^21^ Procarbazine is an oral alkylating agent with primary hematologic toxicity.^22^ Procarbazine is associated with nausea, vomiting, pancytopenia and a 2%‐15% risk of secondary malignancy. Lomustine (CCNU) is an oral alkylating nitrosourea agent, with primary toxicity of myelosuppression and gastrointestinal discomfort. Vincristine is an intravenous microtubule inhibitor with a primary concern of neurotoxicity.^23^, ^24^ Further, there is concern that vincristine adds little clinical benefit given suboptimal CNS penetration observed in animal models.^25^, ^26^, ^27^ One could speculate that vincristine CNS penetration could be higher following radiotherapy, however thus far, there are no data supporting improved passage through the BBB following radiotherapy. A retrospective series of 57 patients who received PCV for LGG showed that the combination of these agents resulted in severe hematologic toxicity (clinically significant grade 3 or higher anemia [7%], neutropenia [10%], and thrombocytopenia [28%]), elevation in liver enzymes (65%), cutaneous rash (26%), neurotoxicity (60%), and vomiting (40%).^28^ In addition to the unfavorable toxicity profile, the rationale for utilizing PCV chemotherapy is based on our highest quality data with the longest follow‐up.

In RTOG 9802, 51% and 15% of patients in the radiotherapy and PCV arm experienced a grade 3 or 4 hematologic toxicity, respectively, compared to 8% and 3% respective values among patients who received radiotherapy alone (*P* < .001).^5^, ^16^ Only half of patients randomized to the chemotherapy arm received the full chemotherapy per protocol; the median number of cycles received was 3 for procarbazine, 4 for CCNU, and 4 for vincristine.^5^ In the EORTC 26 951 trial, only 30% of patients completed the intended 6 cycles of PCV (median 3 cycles received), with premature discontinuation due to hematologic toxicity in 33% of patients and tumor progression in 24% of patients.^18^ In RTOG 9402, only 48% of patients received the intended 4 cycles of dose‐intensive PCV, and the majority of patients discontinuing chemotherapy due to toxicity or progression.^19^

## 4 THE CASE FOR TEMOZOLOMIDE

Temozolomide is an oral alkylating agent introduced in the early 1990s with great enthusiasm given its penetration of the blood‐brain barrier and excellent overall tolerability.^29^, ^30^ Indeed, standard regimen TMZ (150‐200 mg/m^2^ days 1‐5 of a 28‐day cycle) is relatively well‐tolerated compared to PCV chemotherapy, associated with nausea and vomiting that is typically responsive to antiemetic therapy and dose‐limiting grade 3‐4 thrombocytopenia in 7%‐17%.^31^, ^32^, ^33^, ^34^ Further, unlike with nitrosoureas, TMZ is not associated with cumulative hematologic toxicity, with the exception of myelodysplastic syndrome, AML, and ALL.^35^ It is rare for TMZ to cause severe myelosuppression resulting in discontinuation of therapy.^34^

TMZ has an established role in improving survival of patients treated with radiation for glioblastoma. The landmark EORTC‐NCIC trial randomized patients with glioblastoma to receive radiotherapy alone vs radiotherapy with concurrent and adjuvant temozolomide.^36^, ^37^ The initial report revealed a marked 2‐year overall survival advantage in patients treated with concurrent and adjuvant TMZ compared to radiotherapy alone (26.5% vs 10.4%, respectively, *P* < .001).^36^ Moreover, this result was sustained with greater follow‐up, as the 5‐year overall survival for patients treated with combined radiotherapy and TMZ vs radiotherapy alone was 9.8% vs 1.9%, respectively (*P* < .0001).^37^

Temozolomide has also been shown to improve survival compared to radiotherapy alone for patients with non‐1p/19q codeleted anaplastic gliomas.^38^ The CATNON (EORTC 26053‐22054) trial randomized 745 patients with newly diagnosed noncodeleted anaplastic gliomas between 2007 and 2015 to receive radiotherapy (59.4 Gy in 1.8 Gy fractions) with or without adjuvant temozolomide (12 4‐week cycles) or to receive radiotherapy plus concurrent TMZ with or without adjuvant TMZ.^38^ The final report of this study is pending; however, a preplanned interim analysis showed that patients who received 1 year adjuvant TMZ compared to radiotherapy alone exhibited a significant improvement in overall survival (HR 0.65, 99.145% CI: 0.45‐0.93, *P* = .0014).^38^ Recently, the second interim and first molecular analysis of this study was presented at the 2019 meeting of the American Society of Clinical Oncology (ASCO).^39^ In the overall cohort, concurrent administration of TMZ was not associated with improved survival, however there was a trend toward a survival benefit in patients with *IDH*‐mutant tumors treated with concurrent TMZ (5 year OS: 76% with concurrent TMZ vs 68% without, HR 0.63, *P* = .012), however this was not seen among patients with *IDH*‐WT tumors.^39^ In addition, there was a trend toward improved 5 year OS only for *IDH*‐mutant patients receiving adjuvant TMZ but not *IDH*‐WT patients (83% with adjuvant TMZ vs 60% without, HR 0.46, *P* = .012).^39^ A caveat to this report is that this analysis was not conducted in the context of *MGMT* promoter methylation status, which strongly predicts for the survival benefit from TMZ for glioblastoma patients.

Promising results for combined TMZ and radiotherapy have also been seen for patients with high‐risk LGG. RTOG 0424 was a phase II, single‐arm study which enrolled 136 patients with grade II gliomas between 2005 and 2009.^40^ Patients were required to have at least 3 unfavorable factors^6^: age ≥ 40 years, preoperative tumor diameter ≥ 6 cm, bihemispherical tumor, astrocytoma histology, and/or preoperative neurological function status of > 1 (ie, moderate to severe impairment).^40^ The study was designed to detect a 20% improvement in 3‐year overall survival compared to historical controls. Patients received 54 Gy (in 1.8 Gy fractions) with concurrent and adjuvant TMZ for 6 months. The 3‐year OS rate observed was 73.1% which was noted to be significantly higher than prespecified historical control values (*P* < .001), although molecular subtyping was not available.^40^ In terms of toxicity, any grade 3 and 4 events occurred in 43% and 10% of patients, respectively, and one person died from herpes encephalitis.^40^ Grades 3 and 4 hematologic toxicity were 24% and 8%, respectively, which are lower compared to the hematologic toxicity rates associated with the use of PCV in RTOG 9802 where grade 3 and 4 toxicity in radiation therapy and PCV arm was 51% and 15%, respectively.^16^ Although unknown, this may reflect the overall improved treatment and care for these patients, such as more conformal radiation therapy and better supportive care with chemotherapy. The level of evidence of this study is an obvious limitation, as patterns observed from single‐arm phase II trials compared to historical controls do not always hold up when further tested in the randomized phase III setting.

## 5 COMPARISON OF PCV AND TMZ

There are currently no mature data providing a direct comparison of adjuvant PCV vs TMZ in addition to radiotherapy for the upfront treatment of high‐risk LGG. The ALLIANCE‐N0577‐CODEL trial is an ongoing prospective study randomizing patients with LGG to receive adjuvant PCV following radiotherapy vs concurrent and adjuvant TMZ; however, this study is restricted to patients with 1p/19q codeletion. Thus, much of clinical practice requires extrapolation from existing data. Relevant prospective and retrospective studies comparing TMZ with PCV or nitrosourea chemotherapy regimens are reviewed here and summarized in Table 1.

**Table 1:** Relevant prospective and retrospective studies comparing TMZ with PCV/nitrosourea chemotherapy for recurrent or primary LGG

Study	Study type	Patient population	Comparison	Main results	Conclusion/limitations
NOA‐04 Wick et al (2016)	Prospective Randomized Trial	274 Anaplastic gliomas	Upfront PCV vs TMZ Chemotherapy (within larger randomization between upfront RT vs chemo)	Pts with *IDH*‐mutant/1p/19q codeleted tumors had better PFS when PCV given upfront compared to TMZ; trend to better TTF and OS no different but limited number of events	Favors PCV
RTOG 9813 Chang et al (2017)	Prospective Randomized Trial	196 Anaplastic astrocytoma	RT with concurrent and adjuvant nitrosourea (CCNU or BCNU) vs TMZ	No significant improvement in OS or TTP between arms; TMZ better tolerated	Favors TMZ Prematurely stopped due to poor accrual; not PCV
ICR UK Brada et al (2010)	Prospective Randomized Trial	447 Recurrent (chemotherapy naïve) AA or GBM	PCV vs TMZ‐5 vs TMZ‐21	No difference in OS or PFS between PCV and TMZ	Favor TMZ given toxicity; not upfront setting
Lassman et al (2011)	Retrospective	1013 Anaplastic oligodendro‐glial tumors	Chemoradiotherapy, RT alone, chemotherapy (TMZ vs PCV) alone	Median TTP longer following PCV alone compared to TMZ alone in 1p/19q codeleted patients	Favors PCV
Brandes et al (2006)	Retrospective	109 Anaplastic atrocytoma	Adjuvant chemotherapy (PCV vs TMZ) following surgery & RT	No PFS or OS difference between PCV and TMZ chemotherapy	Favor TMZ given toxicity profile; small numbers, retrospective

Abbreviations: AA, anaplastic astrocytoma; GBM, glioblastoma multiforme; LGG, lower‐grade glioma; OS, overall survival; PCV, procarbazine, lomustine, and vincristine; PFS, progression free survival; Pts, patients; TMZ, temozolomide; TTF, time to treatment failure; TTP, time to tumor progression.

Perhaps the best quality data providing some comparison between adjuvant PCV and TMZ is from the German NOA‐04 trial (1999‐2005) that compared the safety and efficacy of upfront single modality radiotherapy or chemotherapy (randomized to PCV or TMZ) with cross over at time of disease progression to chemotherapy (also randomized to PCV or TMZ) or radiotherapy for 274 patients with anaplastic gliomas.^41^, ^42^ In the initial report with median follow‐up of 5.4 years, upfront radiotherapy or chemotherapy with either PCV or TMZ achieved similar PFS and OS.^41^ On subsequent analysis with a longer median follow‐up of 9.5 years, PFS was shown to be significantly longer among patients with 1p/19q codeleted tumors when PCV was given upfront as compared to TMZ (median PFS 9.4 years vs 4.5 years, respectively, HR 0.39, *P* = .031), with a limited number of events for a survival calculation in this group at the time of updated anlaysis.^42^ The authors discuss these findings as the first randomized data to support superiority of PCV alone over TMZ alone in patients with progressive 1p/19q codeleted anaplastic glioma.^42^

Although not a direct comparison of PCV and TMZ, RTOG 9813 was a randomized trial conducted between 2000 and 2007 that compared radiotherapy with concurrent and adjuvant nitrosourea (NU, including CCNU or BCNU) vs concurrent and adjuvant TMZ for the treatment of anaplastic astrocytoma.^43^ Unfortunately, the trial was closed prematurely due to poor accrual, and 97 patients were included in the radiotherapy plus TMZ arm and 99 patients in the radiotherapy plus NU arm. There was no difference between the arms in terms of median survival (3.9 years for the radiotherapy plus TMZ arm vs 3.8 years for the radiotherapy plus NU arm), or time to tumor progression.^43^ Temozolomide was better tolerated compared to NU. Indeed, 60.4% of patients in the TMZ arm completed chemotherapy as planned vs 21.4% in the NU arm, while 47.9% of patients in the TMZ arm experienced a grade 3 or higher side toxicity vs 75.8% in the NU arm (*P* < .001).^43^ The authors concluded that their findings support the use of TMZ over NU for anaplastic astrocytoma given similar outcomes with lower toxicity.^43^

An ICR UK study randomized 447 chemotherapy‐naïve patients with recurrent grade III or IV astrocytomas between 2005 and 2010 to receive PCV or TMZ, with a subrandomization to compare dosing of TMZ‐5 (200mg/m^2^ for 5 days) vs TMZ‐21 (100 mg/m^2^ for 21 days).^44^ For both arms, TMZ cycle was defined as 28 days. With a median follow‐up of 12 months, there was no survival advantage for PCV compared to TMZ, and there was also no PFS benefit of the TMZ arms (combined) compared to PCV. However, median PFS was worse for the PCV arm compared to the TMZ‐5 arm (3.6 months vs 5.0 months, respectively, *P* = .038), but there was no difference of PFS between the PCV arm and the TMZ‐21 arm (4.2 months, *P* = .76).^44^ Compared to TMZ‐21, TMZ‐5 improved overall PFS (HR 1.38, *P* = .023) and there was a trend toward improved OS (HR 1.32, *P* = .056).^44^ Major toxicity was similar across all groups, and the percent of patients completing the full 9 months of treatment was 17%, 26%, and 13% for the PCV, TMZ‐5, and TMZ‐21 groups, respectively.^44^ The authors conclude that either TMZ‐5 or PCV can be recommended as the current standard of care for chemotherapy‐naïve patients with recurrent grade III‐IV astrocytoma, although they note that TMZ‐5 is associated with better initial PFS.^44^ A major limitation for the interpretation of these findings is that the proportion of patients with 1p/19q codeleted tumors is not reported.

EORTC 22 033 is a randomized trial which randomized 477 patients with grade II astrocytoma, oligodendroglioma, or mixed glioma between 2005 and 2012 to receive radiotherapy alone (50.4 Gy) or TMZ alone (75 mg/m^2^ daily for 21 days for a maximum of 8 cycles).^45^ Eligible patients were required to have at least one poor‐risk feature: radiographic progression, new or worsening neurological deficit, tumor size > 5cm, tumor crossing the midline, or age ≥ 40 years. Overall, there was no difference in PFS among patients with LGG treated with either radiotherapy or TMZ chemotherapy alone, however the data are not yet mature for survival analysis.^45^ Exploratory analysis revealed that patients with *IDH*‐mutant, non‐1p/19q codeleted tumors had a longer PFS when treated with radiotherapy alone compared to TMZ alone (HR 1.86, *P* = .0043).^45^ While the study was designed prior to the mature reporting of RTOG 9802, the authors conclude that these findings may support the option of initial TMZ alone for some patients with *IDH*‐mutant 1p/19q codeleted tumors.^45^

A retrospective study of 1013 patients with anaplastic oligodendroglial tumors treated between 1981 and 2007 compared outcomes following treatment with chemoradiotherapy, radiotherapy alone, or chemotherapy alone.^46^ For patients with 1p/19q codeleted tumors, time to progression (TTP) was longer following treatment with combined chemoradiotherapy (median TTP 7.2 years) compared to either chemotherapy alone (median TTP 3.9 years, *P* = .003) or radiotherapy alone (median TTP 2.5 years, *P* < .001).^46^ In addition, the median TTP was longer following PCV alone (7.6 years) compared to TMZ alone (median TTP 3.3 years, *P* = .019) in patients with 1p/19q codeleted tumors.^46^ Although retrospective and encompassing a large timeframe of treatment years, this data set is large with relatively long follow‐up (median follow‐up 5.2 years) and suggests that PCV chemotherapy may be more effective than TMZ.^46^

Finally, another relevant but smaller retrospective series reporting on outcomes of 109 patients with anaplastic astrocytoma who received surgery, radiotherapy, and chemotherapy suggested comparable findings between chemotherapy regimen.^47^ Of 49 patients who received PCV and 60 patients who received TMZ, the 3‐year overall survival rates were 74% and 59%, respectively, and 49% of patients in both groups were progression‐free at 3 years. In terms of toxicity, adjuvant chemotherapy was interrupted for prolonged hematologic and nonhematologic toxicity in 37% of the PCV group compared to 0% of the TMZ group. The authors conclude that their findings favor TMZ over PCV given similar clinical efficacy and reduced toxicity.^47^

## 6 DOES THE NUMBER OF PCV CYCLES RECEIVED MATTER?

It is clear that the toxicity of PCV has the potential to limit delivery of the prescription dose, and based on the studies reviewed above, a significant subset of patients receiving PCV will discontinue therapy early.^10^, ^11^, ^41^, ^47^ However, it is unclear whether cessation of PCV chemotherapy early has an impact on disease outcome.

To address this question, Tabouret et al^48^ conducted a multi‐center retrospective study of 89 patients treated between 2007 and 2011 for histologically confirmed grade II or III oligodendroglial or mixed gliomas who received PCV. For all patients, 6 cycles of PCV were planned with a minimum of one cycle was delivered. PCV was given at relapse in 73% of the cohort. Only 37% of patients completed 6 cycles; PCV was discontinued in 13.4% due to toxicity and the remainder discontinued PCV due to tumor progression. Restricting the analysis to patients who received at least 2 cycles of PCV and who did not exhibit disease progression while on PCV, discontinuation due to toxicity was associated with worse PFS (HR 2.35, *P* = .023) and OS (HR 5.09, *P* = .021).^48^ This study has several limitations. First, the majority of patients in this cohort were treated in the recurrent setting and this finding may not translate to PCV efficacy in the up‐front setting. Furthermore, the study design was retrospective and it is unknown whether the decrement in survival seen is due to discontinuation of PCV due to toxicity or due to another unaccounted‐for factor.

Despite these limitations, the finding of a possible relationship between PCV discontinuation for toxicity and survival has potential clinical implications. First, further prospective studies are needed to establish the minimum amount of PCV required to achieve the survival benefit shown in RTOG 9802, EORTC 26 951, and RTOG 9402. Second, this finding provides the opportunity to consider whether PCV should be avoided in patients for whom a discontinuation of therapy might be expected (eg, poor performance status, elderly patients). Finally, to our knowledge, there have not been reported subgroup analyses of patients enrolled onto RTOG 9802, RTOG 9402, and EORTC 26 951 to assess whether cessation of PCV due to toxicity in these trials had an impact on PFS and OS. These analyses would further inform our understanding of how PCV toxicity may impact survival.

## 7 ONGOING PROSPECTIVE TRIALS AND FUTURE DIRECTIONS

As introduced above, the ALLIANCE‐N0577‐CODEL trial is an ongoing randomized study of patients with 1p/19q codeleted high‐risk lower grade gliomas designed to compare adjuvant PCV chemotherapy with concurrent and adjuvant TMZ.^15^ Inclusion criteria encompass patients with codeleted WHO grade III glioma or codeleted WHO grade II glioma with one or more high‐risk features (age ≥ 40 years, age < 40 with anything less than a gross total resection, documented growth following prior surgery, intractable seizures). The primary endpoint of the study is PFS, and secondary outcomes include OS, time to progression, toxicity, and quality of life. This study, while not expected to mature for another 7‐10 years from now, will provide the highest‐level data needed to address the optimal chemotherapy regimen for use in high‐risk lower grade gliomas (WHO grade II and III) harboring 1p/19q codeletion.

Ultimately, the optimal regimen has yet to be established, as there are ongoing studies to evaluate the efficacy of alkylating agents in combination with novel drugs. Similarly, for MGMT‐methylated glioblastoma, the standard regimen of concurrent and adjuvant TMZ is likely transient until more optimal therapy can be defined. The CeTeg/NOA‐09 trial was a very recently published phase III study of 141 newly diagnosed patients with MGMT‐methylated glioblastoma randomized to receive standard therapy with concurrent and adjuvant TMZ vs experimental therapy with CCNU/TMZ. Patients who received CCNU/TMZ exhibited superior OS compared to the TMZ‐alone (48.1 months vs 31.4 months, *P* = .049).^49^ The standard treatment for glioblastoma patients may change in the very near future as a result of this recent publication. If the future results of the ALLIANCE‐N0577‐CODEL trial eventually provide randomized data to support the use of adjuvant TMZ instead of adjuvant PCV for patients with 1p/19q codeleted lower grade gliomas, then perhaps a future trial should next evaluate outcomes for CCNU/TMZ vs TMZ for this disease as an extrapolation from CeTeg/NOA‐09.

## 8 TREATMENT RECOMMENDATIONS

For patients with high risk grade II and grade III *IDH*‐mutant gliomas regardless of 1p/19q codeletion status, we recommend utilizing adjuvant PCV as in RTOG 9802^5^, ^17^ and RTOG 9402.^20^ There is insufficient evidence to alter this approach based on an estimate of the likelihood that a given patient will be able to complete the full course of 6 cycles, although notably the optimal number of cycles needed to achieve the survival benefit observed in these studies is unknown. For patients with grade II and grade III *IDH*‐mutant gliomas without 1p/19q codeletion who cannot tolerate PCV, adjuvant TMZ is reasonable as supported by CATNON (EORTC 26053‐22054),^38^ given the favorable initial survival benefit reported on interim analysis of this trial. However, it is important to note that the efficacy of radiotherapy and adjuvant PCV for patients with grade II and III *IDH*‐mutant tumors derives from post hoc analyses, whereas the CATNON trial was designed based on molecular inclusion criteria and thus the power of the different trials for influencing treatment recommendations is not the same. Additionally, the subset analysis from RTOG 9802 showing that *IDH*‐mutant high risk grade II gliomas had improved survival with adjuvant PCV compared to RT alone, regardless of 1p/19q status, is also a post hoc analysis that has not yet been published in manuscript form. Nonetheless, we propose that these recommendations represent a balance between known benefit and toxicity. In medically fit patients with *IDH*‐mutant tumors, the large survival benefit of PCV cannot be ignored. In those for whom PCV is considered too toxic, then TMZ is a balanced choice.

## 9 CONCLUSION

In conclusion, while awaiting the initial and mature reporting of CODEL, physicians are faced with the question of whether to administer PCV or the better‐tolerated TMZ to patients with lower grade gliomas. Both approaches (TMZ and PCV) are considered acceptable per guidelines in Europe,^50^ the United States,^51^ and China.^52^ However, despite the widespread use of TMZ, the preponderance of evidence does not support the use of TMZ with radiotherapy as compared to PCV. Ultimately, further investigation is necessary to resolve the question of the optimal chemotherapy regimen for management of patients with lower grade gliomas.

## CONFLICT OF INTEREST

Dr McDuff has nothing to disclose. Dr Dietrich reports “consultant for Monteris and Unum Therapeutics; author for UpToDate.” Dr Atkins has nothing to disclose. Dr Oh has nothing to disclose. Dr Loeffler has nothing to disclose. Dr Shih reports “honoraria from UpToDate; honoraria from Prime Oncology.”
